# Smoking as a Common Modulator of Sensory Gating and Reward Learning in Individuals with Psychotic Disorders

**DOI:** 10.3390/brainsci11121581

**Published:** 2021-11-29

**Authors:** Alexis E. Whitton, Kathryn E. Lewandowski, Mei-Hua Hall

**Affiliations:** 1 Black Dog Institute, University of New South Wales, Sydney, NSW 2031, Australia; 2 Schizophrenia and Bipolar Disorders Program, McLean Hospital, Harvard Medical School, Belmont, Boston, MA 02478, USA; klewandowski@mclean.harvard.edu (K.E.L.); mhall@mclean.harvard.edu (M.-H.H.); 3 Psychosis Neurobiology Laboratory, McLean Hospital, Harvard Medical School, Belmont, Boston, MA 02478, USA

**Keywords:** nicotine, P50, event-related potential, schizophrenia, bipolar disorder

## Abstract

Motivational and perceptual disturbances co-occur in psychosis and have been linked to aberrations in reward learning and sensory gating, respectively. Although traditionally studied independently, when viewed through a predictive coding framework, these processes can both be linked to dysfunction in striatal dopaminergic prediction error signaling. This study examined whether reward learning and sensory gating are correlated in individuals with psychotic disorders, and whether nicotine—a psychostimulant that amplifies phasic striatal dopamine firing—is a common modulator of these two processes. We recruited 183 patients with psychotic disorders (79 schizophrenia, 104 psychotic bipolar disorder) and 129 controls and assessed reward learning (behavioral probabilistic reward task), sensory gating (P50 event-related potential), and smoking history. Reward learning and sensory gating were correlated across the sample. Smoking influenced reward learning and sensory gating in both patient groups; however, the effects were in opposite directions. Specifically, smoking was associated with improved performance in individuals with schizophrenia but impaired performance in individuals with psychotic bipolar disorder. These findings suggest that reward learning and sensory gating are linked and modulated by smoking. However, disorder-specific associations with smoking suggest that nicotine may expose pathophysiological differences in the architecture and function of prediction error circuitry in these overlapping yet distinct psychotic disorders.

## 1. Introduction

Disturbances in motivation and perception are hallmark features of schizophrenia and other psychotic disorders. Although they frequently co-occur within the same individual, these disturbances have been traditionally studied as separate entities. In recent years, however, research emanating from the field of computational neuroscience has postulated predictive coding accounts of psychosis, which cast motivational and perceptual disturbances in a more unified light. Such accounts posit that psychosis results from “a decreased precision in the encoding of prior beliefs relative to the sensory data, thereby garnering maladaptive inferences” [1]; (p. 634). Through this lens, motivational and perceptual disturbances may be conceptualized as arising from faulty prediction and/or prediction error signals. Accordingly, rather than being pathophysiologically distinct, motivational and perceptual disturbances may be governed by a common learning mechanism (for a review, see [2]), perhaps arising from aberrant phasic striatal dopamine signaling [3]. However, to date, empirical studies examining associations between motivational and perceptual disturbances in the same individuals are lacking.

Motivational disturbances, such as anhedonia and avolition, have been linked to aberrant function of brain reward systems [4,5]. Although the nature and extent of reward-processing impairments in psychotic disorders is complex and has been reviewed elsewhere [4,5,6], evidence suggests associations between aberrant reward processing and abnormalities in the neurocircuitry that supports reward prediction error signaling. For example, neuroimaging meta-analyses show that compared to controls, individuals with psychotic disorders exhibit abnormal striatal reward prediction error signals and reduced ventral striatal activation during anticipation of reward [7]. Although the mechanisms underpinning these disturbances are still unclear, findings of abnormally increased striatal dopamine synthesis, storage, and release in individuals with psychosis, relative to healthy controls [8], have led to the hypothesis that elevated levels of striatal dopamine may disrupt the phasic dopamine burst firing that supports prediction errors signals [3,9].

A critical function subserved by striatal reward prediction error signals is reward-based learning, which refers to the ability to adaptively modulate behavior as a function of prior reinforcement. Despite evidence of aberrant reward prediction error signaling in individuals with psychosis, research examining the acquisition of reward-based learning in psychotic disorders, particularly in schizophrenia, has generated debate about the nature and extent of reward learning deficits [10,11]. Within this literature, several studies show impairments in acquisition of reward learning in individuals with schizophrenia [10,12,13,14]. However, others have found no differences in the acquisition of reward-based learning compared to non-psychiatric controls [11,15]). Other studies have shown evidence of deficits in explicit but not implicit reward learning [16], deficits in other aspects of learning that require explicit updating of reward contingencies (e.g., reversal learning) [13,14], as well as more rapid decay of reward-based memory [15]. When examining reward learning in individuals across the psychosis spectrum, studies have also shown that even in the face of nonsignificant group differences, psychotic symptom severity correlated with performance on a measure of reward learning [17]. Taken together, empirical studies are broadly consistent with a disruption in reward-based learning in individuals with psychotic disorders.

Perceptual disturbances frequently co-occur with motivational impairments in psychosis, among the most common of which are auditory hallucinations. Auditory hallucinations have been explained as a failure of corollary discharge mechanisms to distinguish self-generated and externally generated percepts (e.g., [18,19]), and evidence implicates aberrant auditory sensory gating in the pathophysiology of this phenomenon [20,21,22]. The connection suggests that auditory sensory gating deficits, in addition to being a heritable trait [23,24], are sensitive to auditory hallucination states. Disruption of sensory gating is also postulated to reflect a fundamental neural deficit that may contribute to several other core symptom dimensions in psychosis, including positive symptoms, negative symptoms, and cognitive impairments. For example, it has been reported that impaired sensory gating is associated with greater difficulties in attention, poorer working memory, or reduced processing speed [25,26,27,28], although other studies fail to find evidence of these associations [29]. Similarly, sensory gating deficits have been found to be associated with negative symptoms in some [26,30] but not other studies [31]. Interestingly, these symptom dimensions appear to be largely independent of each other, suggesting that a common basic mechanism may underlie several core aspects of psychosis but via separable pathways.

Auditory sensory gating is commonly assessed using a paired-click paradigm involving the presentation of two identical clicks (S1 and S2) in rapid succession. In healthy individuals, inspection of the auditory event-related potential at around 50 ms post-click (the P50 component) typically reveals reduced amplitude in response to S2 relative to S1 [32], interpreted as suppressions of neural responses to redundant auditory information. Meta-analyses indicate that compared to healthy controls, P50 suppression (computed as the ratio of P50 amplitude in response to S2 relative to S1) is attenuated in individuals with schizophrenia, as well as their unaffected family members [24,33], and is also present in individuals with affective psychoses, such as psychotic bipolar disorder [34].

Sensory gating and other aspects of repetition suppression have historically been interpreted as arising from automatic, bottom-up sensory processes [35,36]. However, several studies show that manipulating expectations about the probability of stimulus repetition alters the magnitude of repetition suppression effects in a manner that is not explained by these prior theories. For example, suppression has been found to be stronger when repetition is highly probable and therefore easy to predict, compared to when it is less probable and therefore less predictable [37,38]. When interpreted within a predictive coding framework, smaller neural responses to S2 when repetition is highly predictable occur because the prediction error is minimized [39]. Accordingly, rather than being a purely bottom-up sensory process, sensory gating appears to be influenced by higher-order predictions and learning mechanisms. Recent theories have posited that this form of prediction-based attenuation may be a general property of learning across all neural circuits [40]. An important unanswered question, therefore, is to what extent sensory gating is linked to other aspects of learning (such as reward learning) in the same individuals.

Links between reward learning and sensory gating are also of interest, given that nicotine has been found to improve reward learning [41] and sensory gating [42] in individuals with psychosis. Individuals with schizophrenia exhibit very high rates of nicotine dependence, exceeding those observed in the general population, as well as in other psychiatric disorders [43]. Nicotine is posited to enhance reward learning by amplifying reward-related phasic dopamine burst firing in the striatum [44,45]. Consistent with this, smoking has been found to improve performance on a behavioral reward learning task in individuals with schizophrenia, except when phasic striatal dopamine signaling is blocked by potent dopamine D2 receptor antagonists [41]. Nicotine has also been found to improve sensory gating impairments in individuals with schizophrenia [42,46], with much of the literature focusing on nicotine’s ability to enhance sensory functions via attention-related release of acetylcholine [47]. However, preclinical studies show that phasic dopamine signals are also critical for determining the salience of unexpected sensory stimuli [48] and support the formation of associations between two neutral stimuli, regardless of their reinforcing properties [49,50]. Nicotine may therefore influence sensory gating additionally via dopaminergic mechanisms. Taken together, these studies suggest that reward learning and sensory gating may be linked through predictive coding processes and that nicotine may act as a common modulator of both reward learning and sensory gating via its impact on the dopaminergic neurocircuitry that supports prediction error signaling.

The current study examined whether motivation and perception, measured via reward learning and sensory gating, are linked in individuals with psychotic disorders and whether both are modulated by smoking. We predicted that individuals with psychosis would show impaired reward learning and sensory gating, that these two processes would be correlated, and that smoking would be a common modulator that influences reward learning and sensory gating in a similar way. A second aim was to compare associations in individuals with schizophrenia to individuals with psychotic bipolar disorder. This is an important comparison because although individuals with bipolar disorder share genetic susceptibility with schizophrenia [51], experience perceptual disturbances and P50 sensory gating deficits [24,52], and show high rates of smoking [53], several theoretical models (for reviews, see [4,54,55]) and recent empirical studies [56] suggest a distinct profile of motivational disturbances in bipolar disorder that are potentially attributable to elevated (as opposed to blunted) reward responsiveness. Accordingly, individuals with bipolar disorder may differ from individuals with schizophrenia on important aspects of reward-based prediction and prediction error signaling.

## 2. Materials and Methods

### 2.1. Participants

The sample consisted of 79 patients with schizophrenia-spectrum disorders (i.e., schizophrenia, schizoaffective disorder), 104 patients with bipolar disorder (all had lifetime psychotic features), and 129 healthy controls. All subjects were assessed using the Structured Clinical Interview for DSM-IV [57]. Patients were clinically stable and were recruited from outpatient clinics, flyers posted in a psychiatric hospital, community residential facilities, or by physician referral. The control sample was recruited through local advertisements. All participants had no known neurological disorder, no prior head injury with loss of consciousness, normal hearing confirmed by audiometry, and normal intellectual ability based on the North American Adult Reading Test [58] or years of education (high school completion or more). Patients were included if they had no substance abuse (excluding nicotine) in the preceding 6 months or dependence in the preceding 12 months, and no history of seizures or electroconvulsive therapy in the preceding 12 months. Additional inclusion criteria for controls were: no current or past history of psychotic disorder, bipolar disorder, or schizophrenia-spectrum disorder; no affective disorder (e.g., major depressive disorder) in the preceding 12 months; no substance abuse in the preceding 12 months nor previous chronic dependence; and no first-degree relative with a history of psychosis or bipolar disorder. Controls were all non-smokers. All assessments were conducted in a single visit.

### 2.2. Clinical Symptom Scales

Positive, negative, and general symptoms of psychosis were assessed using the positive and negative syndrome scale (PANSS; [59]); manic symptom severity was assessed using the Young Mania Rating Scale (YMRS; [60]); and depressive symptom severity was assessed using the Montgomery-Asberg Depression Rating Scale (MADRS; [61]). Distinct aspects of negative affective symptoms were measured using the Mood and Anxiety Symptom Questionnaire (MASQ; [62]), which contains subscales for assessing general anxiety, general depression, anxious arousal, and anhedonic depression. Community functioning was assessed using the abbreviated Multnomah Community Ability Scale (MCAS; [63,64]).

### 2.3. Smoking History and Medication

Smoking history was assessed using a questionnaire that asked about number of cigarettes smoked per day, years smoking, and age at which the participant started smoking. Information about psychotropic medication and dosage was obtained from each participant, and chlorpromazine (CPZ) equivalents were calculated based on the recommendations of Baldessarini [65]. In addition to CPZ equivalent, a binary D2 antagonism variable was created by classifying individuals as those who were taking dopamine D2 receptor antagonists (D2 antagonist +) and those who were not taking dopamine D2 receptor antagonists (D2 antagonist −). As described in prior work [41], dopamine D2 receptor antagonists were considered to be any first-generation antipsychotics, in addition to olanzapine and paliperidone.

### 2.4. Sensory Gating

#### 2.4.1. Paired Click Paradigm

Sensory gating was assessed using an auditory paired-click paradigm, as described in our previous studies [23,66]. Auditory stimuli were generated using Presentation software (Neurobehavioral Systems, Inc., Berkeley, CA, USA) and delivered through intra-aural earphones. In total, 160 paired-click stimuli (5 ms duration; 2 ms rise/fall period; 500 ms inter-click interval; 10 s inter-trial interval) were presented in 4 blocks (40 paired-click stimuli per block). Each block was separated by a 1-min rest. Prior to the task, hearing thresholds were ascertained, and stimulus intensity was adjusted to 40 dB above each participant’s hearing threshold.

#### 2.4.2. EEG Recording and Pre-Processing

Each participant was prohibited from smoking for 40 min prior to EEG recordings. The EEG recording and testing procedures followed our previous studies [23,66,67]. The EEG was recorded continuously online using the BioSemi Active Two system (BioSemi Inc, Amsterdam, The Netherland) at a digitization rate of 512 Hz, with a bandpass of DC–104 Hz and a common mode sense as the reference (PO2 site) using an 18-channel electrode cap. Blinks and eye movements were monitored through electrodes placed on the left temple (HEOG) and above and below the left eye (VEOG). The EEG data were re-referenced offline to the averaged mastoid. During the recording, the participants sat comfortably in a chair and relaxed. Participants were asked to fixate on a target on the screen and to minimize their movement and blinking during the presentation of the clicks.

EEG pre-processing was performed offline using NEUROSCAN (4.3) software according to our established methods [23,68]. The continuous EEG from each channel was split into epochs (−100 ms to 400 ms relative to click onset), creating 320 sweeps, and a 10 Hz high-pass filter (24 dB/oct) with zero phase shift was applied. Epochs were then baseline-corrected using the pre-stimulus interval. An automatic ocular artifact-rejection procedure was used to identify artifacts and reject any sweeps ±50 μV in the Cz, HEOG, or VEOG channels from 0–75 ms post-stimulus. Sweeps without artifacts were averaged for each block of 40 trials for the first (S1) and second (S2) clicks separately. Average waveforms were then smoothed by applying a 7-point moving average twice.

#### 2.4.3. Calculation of P50 ERP Components

P50 waveforms were calculated using procedures described previously [23,68]. Briefly, P50 waveforms were measured at Cz. The S1 response was identified as the largest peak from 40 to 80 ms post-stimulus. In accordance with prior work [69,70], the preceding negative trough was used to calculate the S1 amplitude. For the S2 response, the positive peak closest to the S1 peak was selected, and the amplitude was calculated using the same method as was used for calculating the S1 response. The P50 suppression effect was calculated as the ratio (S2/S1) × 100. A higher ratio reflects poorer sensory gating.

### 2.5. Reward Learning

Reward learning was assessed using a well-validated probabilistic reward task (PRT; [71,72]). Grounded in signal detection theory, the PRT provides a measure of a person’s ability to modulate behavior as a function of reinforcement. On each trial of this computerized task, participants were presented with a schematic of a face with two dot eyes. Next, a horizontal straight-line ‘mouth’ was presented quickly (100 ms), and participants were asked to indicate whether the mouth was long (13 mm) or short (11.5 mm). The task comprised three blocks of 100 trials, and on 40% of trials, participants received a monetary reward if they responded correctly (“Correct! You won 20 cents”). Although the long and short mouths were presented at equal frequency across trials, unbeknownst to participants, correct identification of one of the mouth lengths (the ‘rich’ stimulus) was rewarded three times more frequently than correct identification of the other mouth length (the ‘lean’ stimulus). In healthy controls, this asymmetrical reinforcement schedule induces a behavioral response bias toward the rich stimulus [71], and the strength of this bias is hypothesized to indicate an individual’s ability to implicitly learn from positive reinforcement. Prior studies have shown that this response bias is enhanced following exposure to acute nicotine both in healthy controls [73] and in individuals with schizophrenia who are not taking potent dopamine D2 receptor antagonist medications [41]. In addition to the measure of response bias, the PRT also yields a measure of discriminability, which captures the extent to which participants accurately discriminate between the two mouth stimuli. This measure provides a more global measure of stimulus discriminability that is independent of a person’s reward sensitivity.

As in prior studies [71], the PRT data were first subjected to a quality control check, where reaction time outlier trials (<150 ms or >2500 ms) were excluded and where participants who performed below chance level on accuracy (i.e., those with <55% accuracy), had more than 10% outlier trials, or who had a rich:lean reward ratio lower than 2.5:1 (as a result of poor accuracy/slow reaction time) were excluded from analysis. Signal detection analysis [74] was then used to compute response bias and discriminability for each block of the PRT using the following formulae:

(1) $$\mathrm{Response}\;\mathrm{bias}:\;\;\;\;\;\;\;\log\;b=\frac{1}{2}\;\log\left(\frac{{\mathrm{Rich}}_{\mathrm{correct}\;}{*\;\mathrm{Lean}}_{\mathrm{incorrect}}}{{\mathrm{Rich}}_{\mathrm{incorrect}\;}{*\;\mathrm{Lean}}_{\mathrm{correct}}}\right)$$

(2) $$\mathrm{Discriminability}:\;\;\;\;\;\;\;\log\;d=\frac{1}{2}\log\left(\frac{{\mathrm{Rich}}_{\mathrm{correct}\;}{*\;\mathrm{Lean}}_{\mathrm{correct}}}{{\mathrm{Rich}}_{\mathrm{incorrect}\;}{*\;\mathrm{Lean}}_{\mathrm{incorrect}}}\right)$$

To compute response bias and discriminability for cases that had a 0 in the formula, 0.5 was added to each cell of the matrix [75].

### 2.6. Statistical Analyses

Analyses proceeded in four stages. First, we examined differences in sensory gating, response bias, and discriminability between the control, schizophrenia, and bipolar disorder groups using separate analyses of variance (ANOVA). For sensory gating, a one-way ANOVA with Group (control, schizophrenia, bipolar disorder) as the between-subjects factor was used, with the P50 ratio as the dependent variable. For response bias and discriminability, we used a 3 (Group) × 3 (Block: 1, 2, 3) mixed repeated measures ANOVA. Second, we examined associations between sensory gating, response bias and discriminability across the whole sample using Pearson correlations. Third, we examined demographic and clinical characteristics associated with smoking in the schizophrenia and bipolar disorder groups. This was done using separate 2 (Diagnosis: schizophrenia, bipolar disorder) × 2 (Smoking: current smoker, non-smoker) ANOVAs. Finally, we examined the interactive effects of smoking and D2 antagonist medications on sensory gating, response bias, and discriminability in the schizophrenia and bipolar disorder groups. For sensory gating, a 2 (Diagnosis) × 2 (Smoking) × 2 (D2 antagonism: D2 antagonist +, D2 antagonist −) ANOVA was used, with the P50 ratio as the dependent variable. For response bias and discriminability, the effect of Block was also added to the model. Significant ANOVA effects were followed up with Bonferroni-corrected post hoc tests of simple effects.

## 3. Results

### 3.1. Sample Characteristics

The sample comprised 129 non-smoking healthy controls, 79 individuals with schizophrenia (34 smokers, 45 non-smokers), and 104 individuals with bipolar disorder (38 smokers, 66 non-smokers). Demographic and clinical characteristics are shown in Table 1.

### 3.2. Sensory Gating in Patients vs. Controls

**Figure 1:**
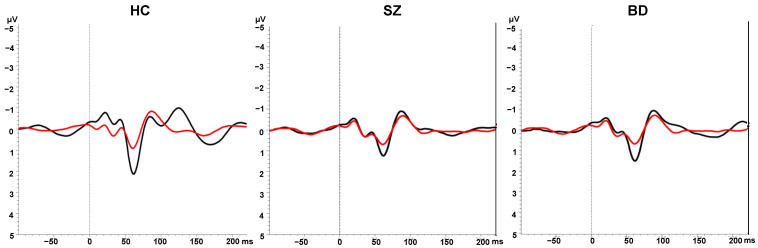
Grand average ERPs in response to S1 (black lines) and S2 (red lines) in the sensory gating paradigm in the healthy control (HC), schizophrenia (SZ), and bipolar disorder (BD) groups.

The average ERP waveforms are shown in Figure 1, and mean P50 ratios are shown in Table 2. A main effect of Group emerged for the P50 ratio, *F*(2, 309) = 35.16, *p* < 0.001, η_p_^2^ = 0.19. Bonferroni-corrected pairwise comparisons showed that compared to the control group, both the schizophrenia (*p* < 0.001) and the bipolar disorder (*p* < 0.001) groups had significantly higher P50 ratios, indicative of poorer sensory gating. The two patient groups did not differ significantly from one another (*p* = 0.64).

### 3.3. Response Bias and Discriminability in Patients vs. Controls

Mean response bias and discriminability in the patient groups and the control group are shown in in Table 2. The 3 (Group) × 3 (Block) ANOVA revealed a main effect of Block, *F*(2, 618) = 6.23, *p* = 0.002, η_p_^2^ = 0.02, where, averaged across groups, response bias in blocks 2 and 3 was significantly higher than in block 1 (both *p*s < 0.05), indicating that the asymmetrical reinforcement schedule successfully induced a behavioral response bias. No significant Group × Block interaction (*p* = 0.42) or main effect of Group (*p* = 0.94) emerged, however, indicating that groups did not differ significantly on reward learning.

A main effect of Block also emerged for discriminability, *F*(2, 614) = 3.80, *p* = 0.02, η_p_^2^ = 0.01, where averaged across groups, discriminability was higher in block 2 than in block 1 (*p* = 0.03). Other pairwise comparisons were not significant (both *p*s > 0.05). There was also a main effect of Group, *F*(2, 307) = 18.41, *p* < 0.001, η_p_^2^ = 0.11. Averaged across blocks, discriminability was significantly lower in the schizophrenia and bipolar disorder groups compared to the control group (both *p*s < 0.001), but the two patient groups did not differ from one another (*p* = 0.79). Taken together, these results indicate that individuals with schizophrenia and bipolar disorder show an impaired ability to learn to discriminate between two visually similar stimuli but perform similarly to controls when learning to acquire a response bias towards a more frequently rewarded visual stimulus.

### 3.4. Associations between Sensory Gating, Response Bias and Discriminability

Across the whole sample, lower P50 ratio (indicative of better sensory gating) was associated with higher overall response bias (*r* = −0.12, *p* = 0.04) and better overall discriminability (*r* = −0.24, *p* < 0.001) across blocks. When examined separately in smokers and non-smokers, correlations between sensory gating and reward learning (*r* = −0.17, *p* = 0.01) and between sensory gating and discriminability (*r* = −0.23, *p* < 0.001) were significant in non-smokers, but not in smokers (both *p*s > 0.2). However, Fisher r-to-z transformation did not show significant differences in the magnitude of correlation strength within these two groups (both *p*s > 0.08), suggesting that the association observed across the whole sample was not conclusively driven by one subgroup.

### 3.5. Demographic and Clinical Features Associated with Smoking in the Patient Sample

A series of 2 (Diagnosis: schizophrenia, bipolar disorder) × 2 (Smoking: Non-smoker, Smoker) ANOVAs were used to assess demographic and clinical characteristics associated with smoking. For brevity, only main effects or interactions involving the Smoking term are reported here. In terms of demographic features, a main effect of Smoking emerged for education, *F*(1, 165) = 9.71, *p* = 0.002, η_p_^2^ = 0.06, where, averaged across the schizophrenia and bipolar disorder groups, smokers had significantly fewer years of education compared to non-smokers. In terms of clinical features, main effects of Smoking also emerged for the PANSS Positive subscale, *F*(1, 172) = 9.04, *p* = 0.003, η_p_^2^ = 0.05, and the YMRS, *F*(1, 172) = 5.75, *p* = 0.02, η_p_^2^ = 0.03, where, averaged across the schizophrenia and bipolar disorder groups, smokers had more severe positive symptoms and more severe manic symptoms compared to non-smokers. A main effect of Smoking also emerged for CPZ equivalent, *F*(1, 174) = 8.15, *p* = 0.005, η_p_^2^ = 0.05, where, averaged across the two patient groups, smokers had a significantly higher psychotropic medication load relative to non-smokers. The Diagnosis × Smoking interaction was not significant for any of the abovementioned demographic or clinical characteristics (all *p*s > 0.05), indicating that the effects of smoking on demographic and clinical features was similar in the schizophrenia and bipolar disorder groups.

### 3.6. Interactive Effects of Diagnosis, Smoking, and D2 Antagonism on Sensory Gating

**Figure 2:**
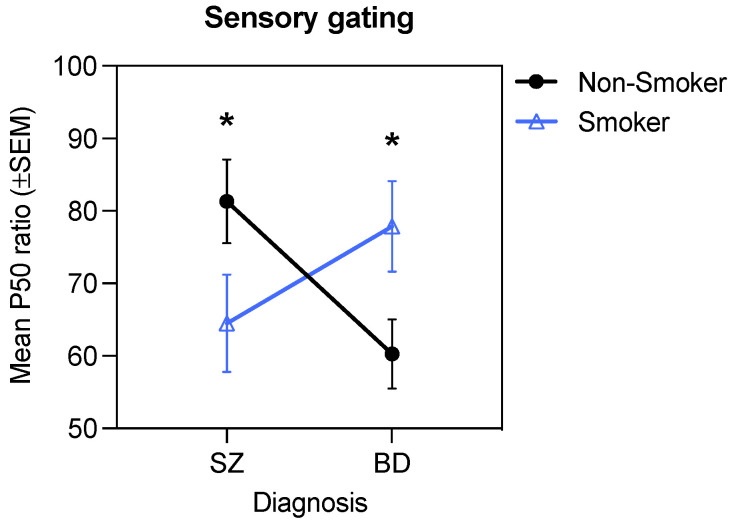
Significant Diagnosis × Smoking interaction for the P50 ratio (* *p* < 0.05).

The 2 (Diagnosis) × 2 (Smoking) × 2 (D2 antagonism: D2 antagonist +, D2 antagonist –) ANOVA for the P50 ratio revealed a significant Diagnosis × Smoking interaction, *F*(1, 172) = 6.12, *p* = 0.01, η_p_^2^ = 0.03 (Figure 2). Post hoc tests of simple effects showed that the P50 ratio differed significantly between the schizophrenia and bipolar disorder groups for non-smokers (*p* = 0.03) but not for smokers (*p* = 0.18), where the P50 ratio was significantly higher in non-smokers with schizophrenia compared to non-smokers with bipolar disorder. There was also a trend for non-smokers with schizophrenia to have higher P50 ratios than smokers with schizophrenia (*p* = 0.09), as well as a trend for smokers with bipolar disorder to have higher P50 ratios than non-smokers with bipolar disorder (*p* = 0.07). There were no main effects or interactions involving D2 antagonism (all *p*s > 0.05). These results indicate that smoking was associated with improved sensory gating in those with schizophrenia but impaired sensory gating in those with bipolar disorder.

### 3.7. Interactive Effects of Diagnosis, Smoking, and D2 Antagonism on Response Bias

**Figure 3:**
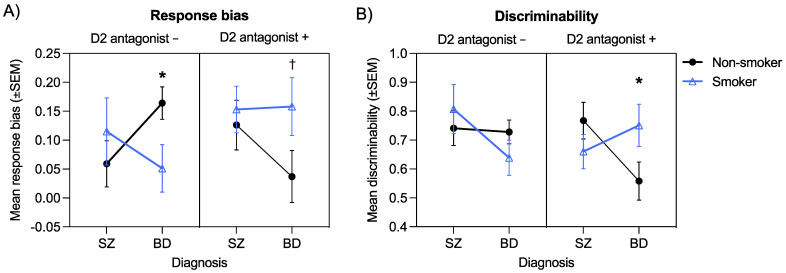
Significant Diagnosis × Smoking × D2 antagonism interaction for response bias (**A**) and discriminability (**B**) (* *p* < 0.05; † *p* < 0.10).

All significant main effects and interactions emerging from the following omnibus ANOVAs are shown in Table 3. A significant Diagnosis × Smoking × D2 antagonism interaction emerged for response bias, *F*(1, 172) = 4.50, *p* = 0.04, η_p_^2^ = 0.03 (Figure 3). This three-way interaction was followed up by examining all possible two-way interactions. For brevity, we focus here on the results of the Diagnosis × Smoking interaction within the D2 antagonist + and D2 antagonist − groups. Results show that the two-way interaction was significant for the D2 antagonist − group, *F*(1, 100) = 3.97, *p* = 0.049, η_p_^2^ = 0.04, but not for the D2 antagonist + group, *F*(1, 72) = 1.07, *p* = 0.31, η_p_^2^ = 0.02. Post hoc tests of simple effects showed that in the D2 antagonist − group, non-smokers with bipolar disorder had significantly higher response bias than smokers with bipolar disorder (*p* = 0.02), whereas there were no differences in response bias between non-smokers and smokers with schizophrenia (*p* = 0.42). Similarly, response bias was significantly higher in non-smokers with bipolar disorder compared to non-smokers with schizophrenia (*p* = 0.03), whereas smokers with bipolar disorder and smokers with schizophrenia did not differ (*p* = 0.36). These findings suggest that in bipolar disorder, smoking enhances response bias in the presence of D2 antagonism but impairs response bias in the absence of D2 antagonism. In contrast, neither smoking nor D2 antagonism impacted response bias in schizophrenia.

### 3.8. Interactive Effects of Diagnosis, Smoking, and D2 Antagonism on Discriminability

A significant Diagnosis × Smoking × D2 antagonism interaction also emerged for discriminability *F*(1, 170) = 6.25, *p* = 0.01, η_p_^2^ = 0.04. As we did for response bias, this three-way interaction was followed up by examining all possible two-way interactions. For brevity, results of the Diagnosis × Smoking interaction within the D2 antagonist + and D2 antagonist − groups are highlighted here. The Diagnosis × Smoking interaction was only significant in the D2 antagonist + group, *F*(1, 72) = 4.45, *p* = 0.04, η_p_^2^ = 0.06. Post hoc tests of simple effects showed that in the D2 antagonist + group, discriminability was higher in non-smokers with schizophrenia compared non-smokers with bipolar disorder (*p* = 0.04), whereas there were no differences in discriminability for smokers with schizophrenia and smokers with bipolar disorder (*p* = 0.37). Similarly, non-smokers with bipolar disorder showed a trend for poorer discriminability than smokers with bipolar disorder (*p* = 0.08), whereas there were no differences between non-smokers and smokers with schizophrenia (*p* = 0.26). These results indicate that smoking enhances discriminability in those with bipolar disorder in the presence of D2 antagonism but does not impact discriminability in the absence of D2 antagonism. No such effects are evident in individuals with schizophrenia.

### 3.9. Evaluation of Potential Confounding Factors

Given that the smoking and non-smoking groups differed on several variables that may have influenced sensory gating and/or reward learning, we conducted further analyses to determine whether these differences may have accounted for our observed effects. First, we assessed possible correlations between sensory gating, reward learning, and the variables on which smokers and non-smokers differed (years of education, medication load, PANSS positive subscale scores, and YMRS scores). Across the whole sample, no significant correlations emerged. However, correlations emerged when examining associations separately in the smoking and non-smoking groups. Specifically, in smokers, education (*r* = −0.25, *p* = 0.04) and medication load (*r* = 0.43, *p* < 0.001) correlated with reward learning. In contrast, in non-smokers, education (*r* = 0.21, *p* = 0.04) and medication load (*r* = 0.27, *p* = 0.005) correlated with sensory gating. Accordingly, we next examined whether the observed interaction effects remained when controlling for education and medication load. The Diagnosis × Smoking interaction for sensory gating remained significant, *F*(1, 153) = 6.53, *p* = 0.01, and the Diagnosis × Smoking × D2 antagonism interaction for reward learning was still at trend level, *F*(1, 153) = 3.10, *p* = 0.08. This suggests that differences in education or medication load between the smoking and non-smoking groups are unlikely to account for our observed effects.

## 4. Discussion

The aim of the current study was to test whether reward learning and sensory gating are linked in individuals with psychotic disorders and whether nicotine acts as a common modulator of these two processes. Replicating prior work, we found that both patient groups had a higher P50 ratio (indicative of poorer sensory gating) compared to controls. We also observed impairments in non-reward-based discriminability on the PRT in both patient groups but normative implicit reward-based learning. Despite the different pattern of group differences observed for the two PRT measures, both showed modest correlations with sensory gating, with better sensory gating correlating with a stronger response bias (*r* = −0.12) and better discriminability (*r* = −0.24) across the sample. Response bias and sensory gating were not significantly correlated with clinical symptoms in the patient group. When considering smoking, we found that smokers and non-smokers differed from one another in a consistent manner across both sensory gating and PRT performance, however, interestingly, opposing effects were observed in the schizophrenia and bipolar disorder groups. Specifically, while smoking was associated with better sensory gating and PRT performance in the schizophrenia group, it was associated with impaired sensory gating and PRT performance in the bipolar disorder group (Figure 3A,B left panel), although the latter impairment was mitigated in individuals with bipolar disorder who were taking dopamine D2 receptor antagonists (Figure 3A,B right panel). Taken together, these findings suggests that sensory gating and aspects of implicit probabilistic learning (both reward-based and reward-independent) are linked across individuals with psychosis and that nicotine may act as a common modulator, albeit with disorder-specific effects.

Of interest is that when collapsed across smokers and non-smokers, sensory gating and PRT performance were similar in individuals with schizophrenia and individuals with bipolar disorder. However, when smoking status was taken into account, inverse patterns of performance were observed in the two disorders, hinting at some degree of mechanistic separation. The direction of effects in smokers relative to non-smokers in each group is resemblant of the inverted U-shaped dose-response relationship posited to account for the variable effects of nicotine on mood and cognition across individuals. A wealth of studies show that the effects of nicotine on mood and cognition are baseline-dependent, with nicotine improving performance in individuals with suboptimal functioning at baseline but impairing performance in individuals with optimal functioning at baseline (for reviews, see [76,77]). The variable effects of smoking observed in the two disorders may therefore arise due to more optimal sensory gating and PRT performance in the bipolar group in the absence of nicotine (i.e., in non-smokers). Studies point to variation in dopamine availability as a factor that may underpin nicotine’s baseline-dependent effects, as the effects of nicotine on sensory gating are moderated by variability in the catechol-O-methyltransferase (COMT) genotype [78] and the dopamine transporter 1 gene [79], both of which are associated with variation in striatal and/or frontal dopamine. Accordingly, although purely speculative, differences in dopaminergic function in schizophrenia and bipolar disorder in the absence of nicotine (i.e., in non-smokers) may account for the opposing effects of smoking on sensory gating and PRT performance in the two groups. Consistent with this idea, evidence of distinct profiles of reward-anticipation-related neural activation in schizophrenia and bipolar disorder suggest that these disorders may indeed be characterized by differences in dopamine function [56].

Evidence that both aspects of PRT performance correlated with the P50 ratio and were modulated by smoking in similar ways supports the notion that rather than being pathophysiologically distinct processes, these processes likely overlap to some extent in terms of their underlying mechanisms. Predictive coding theories [1] and recent integrative models of psychosis emanating from these theories [2,3] provide us with a possible explanation for this link. Predictive coding theory suggests that reward learning and sensory gating are both critically reliant on more general aspects of prediction and prediction error, with recent neurobiological frameworks positioning phasic striatal dopamine firing as the key aberration-driving deficits in prediction error signaling in psychosis [2,3]. This has important implications for how we understand the disparate symptoms of psychosis, like anhedonia and auditory hallucinations. Moreover, it offers a way by which we may be able to target these processes concurrently via novel treatments that can more finely tune striatal dopamine dynamics.

The findings from the current study also raise important questions regarding the neurobiological relevance of smoking across the psychosis spectrum. Although the link between smoking and aspects of cognitive-affective function have been relatively well-studied in schizophrenia, very little is known about how smoking impacts these processes in overlapping yet distinct psychiatric disorders, such as bipolar disorder. This is an important gap, since the pro-cognitive effects of nicotine in schizophrenia have typically been taken as evidence of self-medication via smoking [80], with some noting that similar mechanisms could explain high rates of smoking in bipolar disorder [53]. In recent years, however, the self-medication hypothesis of smoking in schizophrenia has been challenged (e.g., [81,82]) because individuals with schizophrenia do not show greater cognitive gains from smoking or greater cognitive impairments during smoking cessation relative to non-psychiatric healthy controls. Although we observed evidence of slightly better reward learning and sensory gating in individuals with schizophrenia who smoked, the pattern of results in our bipolar disorder group are inconsistent with a self-medication hypothesis. Rather, in those with bipolar disorder, smoking was associated with poorer PRT performance and sensory gating, even though more than one third of the bipolar disorder group smoked. Future research examining the effects of smoking on motivation and perception may reveal important points of neurobiological difference across the psychosis spectrum.

Some limitations must be considered when interpreting our findings. First, we did not compare the effects of acute nicotine to placebo or include a group of psychiatrically healthy smokers in a manner that would be necessary to establish causation. Therefore, the associations between smoking and variability in PRT performance and sensory gating in the current study must be interpreted as correlational only. Relatedly, although we attribute the effects of smoking to the effects of nicotine, it is possible that chronic smoking may result in additional changes in motivation and perception in individuals with psychosis that are distinct from, and which may alter, the acute effects of nicotine. Future randomized controlled studies examining the acute effects of nicotine in smokers and non-smokers are needed to confirm our findings and disentangle nicotine’s acute and chronic effects. Participants were required to abstain from smoking for at least 40 min prior to testing; thus, it is possible that some participants were experiencing the early stages of nicotine withdrawal. The degree to which this may have driven our observed effects is unclear. Additionally, the smoker and non-smoker groups differed on several demographic and clinical variables (education and medication load). Post hoc analyses revealed that education and medication load were associated with reward learning and sensory gating; however, controlling for education and medication load did not change our primary findings (all previous interaction effects were, at minimum, still at trend level). However, future studies with samples better matched on demographic and clinical measures are needed to confirm these findings. Finally, we posit that differences in baseline sensory gating and PRT performance may underpin the divergent effects of smoking observed between the two diagnostic groups, consistent with nicotine’s typical baseline-dependent effects. However, variability in aspects of cognitive functioning (e.g., attention) warrant consideration in future research as an alternative mechanism that may drive our observed effects, particularly given the established links between sensory gating and aspects of cognition.

## 5. Conclusions

In conclusion, our findings indicate that motivation and perception, when assessed via reward learning and sensory gating, are correlated in individuals with psychotic disorders and are modulated by smoking, with opposing effects observed in individuals with schizophrenia and individuals with bipolar disorder. These findings pave the way for future studies into the shared mechanisms that may drive motivational and perceptual impairments in psychotic disorders, as well as the degree to which nicotine can expose pathophysiological differences in the architecture and function of prediction error circuitry in overlapping yet distinct clinical disorders.

## Figures and Tables

**Table 1:** Demographic and clinical characteristics of study cohort.

	Control	Schizophrenia		Bipolar Disorder	
	Non-Smoker (*n* = 129)	Non-Smoker (*n* = 45)	Smoker (*n* = 34)	Non-Smoker (*n* = 66)	Smoker (*n* = 38)
Age	32.02 (12.13)	43.44 (13.45)	41.65 (12.49)	40.14 (13.46)	41.47 (13.27)
Female, *N* (%)	81 (62.8)	17 (37.8)	6 (17.6)	37 (56.1)	19 (50.0)
Yrs. Educ.	15.96 (2.08)	14.86 (2.05)	13.94 (1.94)	15.68 (1.96)	14.69 (1.86)
CPZ equiv.	-	460.23 (446.00)	661.79 (595.40)	161.39 (240.96)	317.63 (368.41)
D2 antag., *N* (%) ^a^	-	20 (46.5)	23 (67.6)	18 (27.3)	15 (40.5)
PANSS Negative	-	10.57 (9.90)	10.26 (8.58)	7.49 (5.78)	8.71 (6.51)
PANSS Positive	-	10.95 (9.42)	14.94 (10.90)	10.24 (8.88)	15.34 (10.38)
PANSS General	-	20.93 (16.88)	23.91 (16.43)	21.98 (15.88)	25.00 (14.43)
YMRS	-	5.59 (7.99)	10.15 (10.10)	8.25 (12.61)	12.23 (13.89)
MADRS	-	11.23 (12.21)	12.15 (12.67)	10.98 (12.43)	10.91 (10.22)
MASQ GDA	-	21.71 (8.44)	22.03 (8.42)	20.77 (8.22)	20.75 (5.66)
MASQ GDD	-	27.69 (11.85)	24.29 (11.41)	25.36 (11.07)	26.58 (10.64)
MASQ AA	-	27.67 (11.10)	28.41 (11.29)	25.53 (9.43)	24.31 (7.64)
MASQ AD	-	66.84 (16.28)	62.09 (18.80)	60.65 (17.43)	65.50 (16.82)
MCAS	-	27.31 (22.29)	29.76 (21.27)	33.95 (22.99)	32.78 (22.14)
Years smoking	-	-	21.38 (13.72)	-	19.29 (13.68)
Cigarettes/day	-	-	10.62 (14.88)	-	10.73 (12.90)

Note: Values are in the format Mean (SD) unless otherwise noted. CPZ equiv. = chlorpromazine equivalent; PANSS = positive and negative symptom scale; YMRS = Young Mania Rating Scale; MADRS = Mood and Anxiety Disorder Rating Scale; MASQ = Mood and Anxiety Symptom Questionnaire; GDA = General Distress Anxiety subscale; GDD = General Distress Depression subscale; AA = Anxious Arousal subscale; AD = Anhedonic Depression subscale; MCAS = Multnomah Community Assessment Scale. ^a^ Medication data for 2 non-smokers with schizophrenia and 1 smoker with bipolar disorder were missing.

**Table 2:** P50 sensory gating, response bias, and discriminability.

	Control	Schizophrenia		Bipolar Disorder	
	Non-Smoker (*n* = 129)	Non-Smoker (*n* = 45)	Smoker (*n* = 34)	Non-Smoker (*n* = 66)	Smoker (*n* = 38)
*Sensory gating, M (SD)*					
P50 ratio	37.48 (25.84)	80.73 (43.37)	63.37 (34.97)	60.91 (28.60)	77.45 (46.01)
P50 S1 amp	3.32 (1.56)	2.48 (1.05)	2.66 (1.66)	3.20 (1.27)	2.79 (1.47)
P50 S2 amp	1.20 (0.91)	1.85 (1.06)	1.49 (0.82)	1.84 (0.93)	1.92 (1.42)
*Response bias, M (SD)*					
Block 1	0.10 (0.25)	0.09 (0.26)	0.13 (0.20)	0.06 (0.21)	0.06 (0.20)
Block 2	0.14 (0.24)	0.09 (0.30)	0.16 (0.24)	0.16 (0.27)	0.08 (0.19)
Block 3	0.14 (0.27)	0.12 (0.29)	0.14 (0.26)	0.16 (0.27)	0.15 (0.27)
Total	0.12 (0.19)	0.09 (0.21)	0.14 (0.19)	0.13 (0.18)	0.09 (0.18)
*Discriminability, M (SD)*					
Block 1	0.89 (0.29)	0.73 (0.33)	0.64 (0.28)	0.65 (0.25)	0.65 (0.29)
Block 2	0.88 (0.29)	0.76 (0.38)	0.74 (0.37)	0.72 (0.34)	0.70 (0.29)
Block 3	0.89 (0.30)	0.74 (0.36)	0.75 (0.39)	0.67 (0.25)	0.69 (0.29)
Total	0.87 (0.25)	0.73 (0.32)	0.69 (0.29)	0.67 (0.23)	0.67 (0.25)

**Table 3:** ANOVA results showing interactive effects of diagnosis (schizophrenia, bipolar disorder), smoking status, and D2 antagonists on P50 ratio, response bias, and discriminability.

	P50 Ratio				Response Bias				Discriminability			
	*df*	*F*	*p*	η_p_^2^	*df*	*F*	*p*	η_p_^2^	*df*	*F*	*p*	η_p_^2^
*Between-subjects effects*												
Diagnosis	1	0.15	0.70	0.001	1	0.12	0.73	0.001	1	2.71	0.10	0.02
Smoking	1	0.003	0.96	<0.001	1	0.54	0.46	0.003	1	0.12	0.73	0.001
D2 antag.	1	0.78	0.38	0.005	1	0.48	0.49	0.003	1	0.97	0.33	0.006
Diagnosis × Smoking	1	6.12	0.01	0.03	1	0.36	0.55	0.002	1	0.64	0.43	0.004
Diagnosis × D2 antag.	1	2.05	0.16	0.01	1	1.04	0.31	0.006	1	0.12	0.73	0.001
Smoking × D2 antag.	1	0.002	0.97	<0.001	1	2.69	0.10	0.02	1	0.36	0.55	0.002
Diagnosis × Smoking × D2 antag.	1	0.74	0.39	0.004	1	4.50	0.04	0.03	1	6.25	0.01	0.04
Error	172				172				170 ^a^			
*Within-subjects effects*												
Block					2	2.74	0.07	0.02	2	5.90	0.003	0.03
Diagnosis × Block					2	0.58	0.56	0.003	2	0.73	0.48	0.004
Smoking × Block					2	0.05	0.95	<0.001	2	1.84	0.16	0.01
D2 antag. × Block					2	1.98	0.14	0.01	2	1.25	0.29	0.007
Diagnosis × Smoking × Block					2	0.72	0.49	0.004	2	1.01	0.35	0.006
Diagnosis × D2 antag. × Block					2	0.31	0.73	0.002	2	0.15	0.86	0.001
Smoking × D2 antag. × Block					2	0.88	0.42	0.005	2	1.74	0.18	0.01
Diagnosis × Smoking × D2 antag. × Block					2	1.13	0.35	0.007	2	1.86	0.16	0.01
Error (Block)					344				340			

^a^ Block 1 discriminability values could not be computed for two subjects.

## Data Availability

The ethical requirement to ensure patient confidentiality precludes public archiving of our data. Researchers who would like to access to raw data should contact the corresponding author, who will liaise with the IRB that approved the study, and as much data as is required to reproduce the results will be released to the individual researcher.
